# The relationship between gut microbiota and COVID-19 progression: new insights into immunopathogenesis and treatment

**DOI:** 10.3389/fimmu.2023.1180336

**Published:** 2023-05-02

**Authors:** Man Wang, Yuan Zhang, Chunmei Li, Wenguang Chang, Lei Zhang

**Affiliations:** ^1^ Institute for Translational Medicine, The Affiliated Hospital of Qingdao University, College of Medicine, Qingdao University, Qingdao, China; ^2^ Department of Radiology, Qingdao Municipal Hospital, Qingdao, China

**Keywords:** COVID-19, gut microbiota, cytokine storm, inflammation, adaptive immunity, microbiota-targeted interventions

## Abstract

The coronavirus disease 2019 (COVID-19) pandemic caused by severe acute respiratory syndrome coronavirus 2 (SARS-CoV-2) has posed a global health crisis. Increasing evidence underlines the key role of competent immune responses in resisting SARS-CoV-2 infection and manifests the disastrous consequence of host immune dysregulation. Elucidating the mechanisms responsible for deregulated host immunity in COVID-19 may provide a theoretical basis for further research on new treatment modalities. Gut microbiota comprises trillions of microorganisms colonizing the human gastrointestinal tract and has a vital role in immune homeostasis and the gut-lung crosstalk. Particularly, SARS-CoV-2 infection can lead to the disruption of gut microbiota equilibrium, a condition called gut dysbiosis. Due to its regulatory effect on host immunity, gut microbiota has recently received considerable attention in the field of SARS-CoV-2 immunopathology. Imbalanced gut microbiota can fuel COVID-19 progression through production of bioactive metabolites, intestinal metabolism, enhancement of the cytokine storm, exaggeration of inflammation, regulation of adaptive immunity and other aspects. In this review, we provide an overview of the alterations in gut microbiota in COVID-19 patients, and their effects on individuals’ susceptibility to viral infection and COVID-19 progression. Moreover, we summarize currently available data on the critical role of the bidirectional regulation between intestinal microbes and host immunity in SARS-CoV-2-induced pathology, and highlight the immunomodulatory mechanisms of gut microbiota contributing to COVID-19 pathogenesis. In addition, we discuss the therapeutic benefits and future perspectives of microbiota-targeted interventions including faecal microbiota transplantation (FMT), bacteriotherapy and traditional Chinese medicine (TCM) in COVID-19 treatment.

## 1 Introduction

The coronavirus disease 2019 (COVID-19), an infectious disease caused by severe acute respiratory syndrome coronavirus 2 (SARS-CoV-2), has been a global pandemic for 3 years (1). Due to reduced viral pathogenicity and widespread immunity from vaccination or natural infection, SARS-CoV-2 becomes endemic in many countries around the world (2). SARS-CoV-2 is likely to constantly circulate among the human population causing an endemic seasonal respiratory infection (3). SARS-CoV-2 is transmitted among human populations through respiratory droplets, aerosols and, to a lesser extent, *via* the faecal-oral route (4). The clinical presentation of COVID-19 ranges from an asymptomatic form, mild respiratory tract illness to severe pneumonia, multiorgan dysfunction, and even death (5). It is acknowledged that the respiratory system is the primary target of SARS-CoV-2 infection (6). Therefore, the common initial manifestations of COVID-19 are respiratory symptoms such as cough and dyspnea (7). Remarkably, human enterocytes with high expression of angiotensin-converting enzyme 2 (ACE2), an entry receptor for SARS-CoV-2, serve as a site of extrapulmonary viral infection as evidenced by the presence of SARS-CoV-2 RNA in faecal samples from COVID-19 patients even after clearance of viral respiratory infection (8, 9). Moreover, gastrointestinal symptoms such as diarrhoea, nausea and vomiting are common in COVID-19 patients (10). COVID-19 patients with gastrointestinal symptoms are prone to develop severe diseases (e.g., liver injury and acute respiratory distress syndrome (ARDS)) and have poorer clinical outcomes (11). Accordingly, SARS-CoV-2 infection-induced gastrointestinal manifestations have recently gained much attention. It is speculated that dysregulated gut microbiota may contribute to the gastrointestinal symptoms in COVID-19 patients.

Gut microbiota refers to a complex population of microbial communities (e.g., bacteria, fungi, protozoa and viruses) that inhabit the mammalian gastrointestinal tract (12). It has many pivotal functions including nutrient acquisition, metabolism, maintenance of intestinal epithelial barrier, modulation of host immunity and protection against diseases (13). Perturbations of gut microbiota, termed gut dysbiosis, are correlated with the pathogenesis of various diseases, including allergy, cancer, inflammatory bowel disease, metabolic syndrome and obesity (14). The composition of gut microbiota is altered in SARS-CoV-2-infected individuals, alluding to the linkage between intestinal microbes and COVID-19 development (15, 16). With more and more scientific evidence and research reports, the underlying mechanisms are gradually emerging. In this review, we provide an overview of correlations between gut microbiota dysbiosis and SARS-CoV-2 pathogenicity. We also summarize the mechanisms responsible for the implication of gut microbiota in COVID-19 progression. In addition, we highlight manipulation of gut microbiota as a therapeutic approach for COVID-19 treatment.

## 2 Dynamic alterations of gut microbiota during SARS-CoV-2 infection

The gut microbiota of COVID-19 patients was seriously dysbiotic, characterized by an expansion of conditional pathogens (e.g., *Actinomyces*, *Akkermansia*, *Collinsella*, *Enterococcus*, *Lactobacillus*, *Lactococcus*, *Methanobrevibacter*, *Odoribacter*, *Parabacteroides*, *Phascolarctobacterium*, *Serratia* and *Staphylococcus*) and a reduction in the relative abundance of beneficial microbes (e.g., *Bacteroides*, *Blautia*, *Coprococcus*, *Dialister*, *Faecalibacterium*, *Lachnospira*, *Oscillospira*, *Prevotella*, *Roseburia*, *Ruminococcus*) (17) (**Table 1**). Notably, *Enterococcus* might be clinically associated with illness severity due to its pathogenic capacity. Gut microbiota of COVID-19 patients serves as a reservoir of opportunistic pathogens that may migrate across impaired epithelial barriers into circulation. Secondary *Enterococcus* infection could be a non-negligible complication associated with poor outcomes in COVID-19 (29). Future research studies are required to delve into whether SARS-CoV-2 infection acts as a predisposing factor to secondary pathogen infections. It is necessary to understand whether coinfection of SARS-CoV-2 and *Enterococcus* leads to worse clinical outcomes in COVID-19 patients. Another study showed that the opportunistic microorganisms *Enterococcus faecium*, *Escherichia coli*, *Klebsiella pneumoniae*, *Salmonella enterica* and *Staphylococcus auricularis* were enriched in COVID-19 patients while the probiotic bacteria *Bacteroides vulgatus*, *Eubacterium eligens* and *Faecalibacterium prausnitzii* were reduced compared with healthy controls (20). It was found that *Acinetobacter* and *Rhodococcus* were negatively correlated with the levels of CD3, CD4, CD45, haemoglobins and red blood cells (RBC) (**Table 2**). *Bacteroides* and *Veillonella* had a positive relationship with CD3, haemoglobins and RBC. *Enterococcus* was positively associated with the plasma concentration of carbon dioxide. These results suggested that gut microbiota alterations might be connected with the immune status and clinical manifestations of inflammation in COVID-19 patients. Nevertheless, the clinical implications of intestinal microbiota deserve further research.

**Table 1:** Gut microbiota profiles of SARS-CoV-2-infected subjects.

Microorganism	Phylum	Family	Relative abundance (SARS-CoV-2-infected subjects vs. healthy controls)	Reference
Pathogenic microbes				
*Actinomyces*	Actinomycetota	Actinomycetaceae	Enriched	(17–19)
*Akkermansia*	Verrucomicrobiota	Akkermansiaceae	Enriched	
*Collinsella*	Actinomycetota	Coriobacteriaceae	Enriched	
*Enterococcus*	Firmicutes	Enterococcaceae	Enriched	
*Lactobacillus*	Firmicutes	Lactobacillaceae	Enriched	
*Lactococcus*	Firmicutes	Streptococcaceae	Enriched	
*Methanobrevibacter*	Euryarchaeota	Methanobacteriaceae	Enriched	
*Odoribacter*	Bacteroidetes	Odoribacteraceae	Enriched	
*Parabacteroides*	Bacteroidetes	Tannerellaceae	Enriched	
*Phascolarctobacterium*	Firmicutes	Acidaminococcaceae	Enriched	
*Serratia*	Pseudomonadota	Yersiniaceae	Enriched	
*Staphylococcus*	Firmicutes	Staphylococcaceae	Enriched	
*Enterococcus faecium*	Firmicutes	Enterococcaceaes	Enriched	(20)
*Escherichia coli*	Pseudomonadota	Enterobacteriaceae	Enriched	
*Klebsiella pneumoniae*	Pseudomonadota	Enterobacteriaceae	Enriched	
*Salmonella enterica*	Pseudomonadota	Enterobacteriaceae	Enriched	
*Staphylococcus auricularis*	Firmicutes	Staphylococcaceae	Enriched	
*Acinetobacter*	Pseudomonadota	Moraxellaceae	Enriched	
*Rhodococcus*	Actinomycetota	Nocardiaceae	Enriched	
*Dorea*	Firmicutes	Lachnospiraceae	Enriched	(18, 19, 21)
*Streptococcus*	Firmicutes	Streptococcaceae	Enriched	
*Bacteroides*	Bacteroidetes	Bacteroidaceae	Enriched	(19, 21, 22)
*Corynebacterium*	Actinomycetota	Corynebacteriaceae	Enriched	
*Enterocloster*	Firmicutes	Lachnospiraceae	Enriched	
*Flavonifractor*	Firmicutes	Oscillospiraceae	Enriched	
*Collinsella aerofaciens*	Actinomycetota	Coriobacteriaceae	Enriched	(23)
*Collinsella tanakaei*	Actinomycetota	Coriobacteriaceae	Enriched	
*Morganella morganii*	Pseudomonadota	Morganellaceae	Enriched	
*Streptococcus infantis*	Firmicutes	Streptococcaceae	Enriched	
*Ruminococcus gnavus*	Firmicutes	Lachnospiraceae	Enriched	
*Clostridium hathewayi*	Firmicutes	Clostridiaceae	Enriched	
*Enterococcus avium*	Firmicutes	Enterococcaceae	Enriched	
Prevotellaceae	Bacteroidetes	–	Enriched	(24)
*Clostridium aldenense*	Firmicutes	Lachnospiraceae	Enriched	(25, 26)
*Escherichia unclassified*	Pseudomonadota	Enterobacteriaceae	Enriched	
*Intestinibacter bartlettii*	Firmicutes	Peptostreptococcaceae	Enriched	
*Clostridium bolteae*	Firmicutes	Lachnospiraceae	Enriched	
*Flavonifractor plautii*	Firmicutes	Oscillospiraceae	Enriched	
*Clostridium ramosum*	Firmicutes	Clostridiaceae	Enriched	
*Candida albicans*	Ascomycota	Debaryomycetaceae	Enriched	(27, 28)
Beneficial microbes				
*Blautia*	Firmicutes	Lachnospiraceae	Depleted	(17, 20–22, 24)
*Coprococcus*	Firmicutes	Lachnospiraceae	Depleted	
*Dialister*	Firmicutes	Veillonellaceae	Depleted	
*Faecalibacterium*	Firmicutes	Oscillospiraceae	Depleted	
*Lachnospira*	Firmicutes	Lachnospiraceae	Depleted	
*Oscillospira*	Firmicutes	Oscillospiraceae	Depleted	
*Prevotella*	Bacteroidetes	Prevotellaceae	Depleted	
*Roseburia*	Firmicutes	Lachnospiraceae	Depleted	
*Ruminococcus*	Firmicutes	Oscillospiraceae	Depleted	
*Bacteroides vulgatus*	Bacteroidetes	Bacteroidaceae	Depleted	(20, 26)
*Eubacterium eligens*	Firmicutes	Eubacteriaceae	Depleted	
*Faecalibacterium prausnitzii*	Firmicutes	Clostridia	Depleted	
*Veillonella*	Firmicutes	Veillonellaceae	Depleted	
*Megasphaera*	Firmicutes	Veillonellaceae	Depleted	(21)
*Alistipes onderdonkii*	Bacteroidetes	Rikenellaceae	Depleted	(23)
*Bacteroides stercoris*	Bacteroidetes	Bacteroidaceae	Depleted	
*Lachnospiraceae bacterium 1_1_57FAA*	Firmicutes	Lachnospiraceae	Depleted	
*Parabacteroides merdae*	Bacteroidetes	Tannerellaceae	Depleted	
*Bacteroides uniformis*	Bacteroidetes	Bacteroidaceae	Depleted	
*Parabacteroides distasonis*	Bacteroidetes	Tannerellaceae	Depleted	
*Adlercreutzia*	Actinomycetota	Eggerthellaceae	Depleted	(22, 24)
Bacteroidaceae	Bacteroidetes	–	Depleted	
*Eubacterium brachy*	Firmicutes	Eubacteriales Family XIII. Incertae Sedis	Depleted	
Lachnospiraceae	Firmicutes	–	Depleted	
Ruminococcaceae	Firmicutes	–	Depleted	
*Butyricicoccus pullicaecorum*	Firmicutes	Clostridiaceae	Depleted	(25, 26)
*Eubacterium hallii*	Firmicutes	Lachnospiraceae	Depleted	
*Fusicatenibacter saccharivorans*	Firmicutes	Lachnospiraceae	Depleted	
*Intestinimonas butyriciproducens*	Firmicutes	–	Depleted	
*Roseburia inulinivorans*	Firmicutes	Lachnospiraceae	Depleted	
*Ruminococcus bromii*	Firmicutes	Oscillospiraceae	Depleted	
Firmicutes	–	–	Depleted	(22)
*Aspergillus*	Ascomycota	Aspergillaceae	Depleted	(27)
*Candida parapsilosis*	Ascomycota	Debaryomycetaceae	Depleted	
*Malassezia yamatoensis*	Basidiomycota	Malasseziaceae	Depleted	
*Moesziomyces aphidis*	Basidiomycota	Ustilaginaceae	Depleted	
*Mucor racemosus*	Mucoromycota	Mucoraceae	Depleted	
*Penicillium citrinum*	Ascomycota	Aspergillaceae	Depleted	
*Penicillium polonicum*	Ascomycota	Aspergillaceae	Depleted	
*Rhodotorula mucilaginosa*	Basidiomycota	Sporidiobolaceae	Depleted	
*Talaromyces wortmannii*	Ascomycota	Trichocomaceae	Depleted	
*Trechispora* sp.	Basidiomycota	Hydnodontaceae	Depleted	
*Wallemia sebi*	Basidiomycota	Wallemiaceae	Depleted	

**Table 2:** Effects of dysregulated gut microbiota on the immune status and COVID-19 progression.

Microbial change	Potential effect	Outcome	Reference
↑*Acinetobacter*, *Rhodococcus*	Inversely correlate with CD3, CD4, CD45, haemoglobins and RBC	Promote COVID-19 severity	(20)
↓*Bacteroides*, *Veillonella*	Positively correlate with CD3, haemoglobins and RBC	Lessen COVID-19 severity	
↓*Enterococcus*	Positively correlate with the plasma concentration of carbon dioxide	Lessen COVID-19 severity	
↑*Eubacterium dolichum*, *Prevotella copri*	Positively correlate with SARS-CoV-2 viral load	Promote COVID-19 severity	(30)
↓*Alistipes*, *Bifidobacterium*, *Clostridium citroniae*, *Dialister*, *Haemophilus*, *Haemophilus parainfluenzae*, *Ruminococcus*, *Streptococcus anginosus*	Inversely correlate with SARS-CoV-2 viral load	Lessen COVID-19 severity	
↑*Actinomyces viscosus*, *Bacteroides nordii*, *Clostridium hathewayi*	Induce bacteremia	Promote COVID-19 severity	(31)
↑*Coprobacillus*, *Clostridium hathewayi*, *Clostridium ramosum*	Positively correlate with COVID-19 severity	Promote COVID-19 severity	
↓*Alistipes onderdonkii*, *Faecalibacterium prausnitzii*	Inversely correlate with COVID-19 severity	Lessen COVID-19 severity	
↑*Alistipes finegoldii*, *Clostridium innocuum*, *Ruthenibacterium lactatiformans*	Positively correlate with inflammatory biomarkers CRP and WBC	Promote COVID-19 severity	(32)
↓*Alistipes putredinis*, *Blautia luti*, *Dorea longicatena*, *Faecalibacterium prausnitzii*, *Gemmiger formicilis*	Inversely correlate with COVID-19 severity	Lessen COVID-19 severity	
↑*Bacteroides*	Positively correlate with COVID-19 severity	Promote COVID-19 severity	(33)
↓*Bifidobacterium*, *Roseburium*	Inversely correlate with COVID-19 severity	Lessen COVID-19 severity	
↑*Aspergillus*	Positively correlate with COVID-19 severity	Promote COVID-19 severity	(34)
↑*Blautia*, *Lactobacillus*, *Ruminococcus*	Positively correlate with proinflammatory cytokines IFN-γ, IL-2, IL-4, IL-6, IL-8, IL-10 and TNF-α	Magnify inflammation	(35)
↓*Bacteroides*, *Clostridiales*, *Streptococcus*	Inversely correlate with proinflammatory cytokines IFN-γ, IL-2, IL-4, IL-6, IL-8, IL-10 and TNF-α	Inhibit inflammation	
↑*Burkholderia contaminans*	Positively correlate with inflammation biomarkers CRP and IL-6; inversely correlate with the levels of lymphocytes, CD3^+^ T cells and CD4^+^ T cells	Magnify inflammation; impair adaptive immune responses	(36)
↑*Enterococcus faecalis*	Inversely correlate with the count of CD8^+^ T cells	Impair adaptive immune responses	(37)
↓*Lachnospira*, *Prevetolla*, *Roseburia*	Inversely correlate with IL-21	Inhibit inflammation	(38)
↑*Akkermansia muciniphila*, *Bacteroides dorei*	Positively correlate with IL-6, CXCL8 and IL-1β	Magnify inflammation	(39)
↓*Bifidobacterium adolescentis*, *Collinsella aerofaciens*, *Coprococcus comes*, *Dorea longicatena*, *Eubacterium rectale*, *Faecalibacterium prausnitzii*	Inversely correlate with CXCL10	Inhibit inflammation	
↓*Collinsella aerofaciens*, *Coprococcus comes*, Dorea formicigenerans, *Dorea longicatena*, *Ruminoccocus obeum*	Inversely correlate with IL-10	Inhibit inflammation	
↓*Collinsella aerofaciens*, *Coprococcus comes*	Inversely correlate with TNF-α	Inhibit inflammation	
↓*Coprococcus comes*, *Eubacterium rectale*	Inversely correlate with CCL2	Inhibit inflammation	
↓Clostridia	Inversely correlate with IFN-γ	Inhibit inflammation	(40)
↑Actinobacteria	Positively correlate with the gp130/sIL-6Rb level	Magnify inflammation	
↑*Citrobacter*, *Fusobacterium, Peptostreptococcus*	Positively correlate with faecal IL-18 level	Facilitate the cytokine storm	(41)
↑*Enterococcus faecalis* (GroEL)	Positively correlate with IL-6 and IL-10	Facilitate the cytokine storm	(37)
↑*Eubacterium ramulus*	Inversely correlate with IL-6	Prevent the cytokine storm	
↑*Blautia obeum*, *Coprococcus catus*, *Coprococcus comes*	Positively correlate with the number of lymphocytes, CD3^+^ T cells, CD4^+^ T cells and CD8^+^ T cells and lymphocyte proportion	Enhance adaptive immune responses	(42)
↓*Roseburia intestinalis*	Positively correlate with the number of lymphocytes, CD3^+^ T cells, CD4^+^ T cells and CD8^+^ T cells and lymphocyte proportion	Enhance adaptive immune responses	

↑ represents high abundance; ↓ represents low abundance.

Gut microbiota dysbiosis causes changes in the gut metabolome profiles. Increased abundance of *Blautia*, *Dorea*, *Parabacteroides* and *Streptococcus* resulted in the upregulation of glycerophospholipid, glycerolipid, linoleic acid and ether lipid metabolism in SARS-CoV-2-infected individuals (18). Lipids could be crucial for viral replication and dissemination (43). These identified bacteria may be potential predictors for COVID-19 severity. Gut microbiota diversity was markedly reduced in COVID-19 patients compared with healthy controls (19). The decreased microbial diversity was associated with poor prognosis in COVID-19 patients. COVID-19 patients exhibited an enrichment of opportunistic pathogenic species including *Bacteroides*, *Corynebacterium*, *Enterocloster*, *Enterococcus*, *Flavonifractor*, *Parabacteroides* and *Streptococcus* compared with healthy controls. The abundance of anti-inflammatory butyrate-producing bacteria (e.g., *Dialister*, *Faecalibacterium*, *Lachnospira*, *Megasphaera*, *Prevotella*, *Roseburia* and *Ruminococcus*) was reduced in COVID-19 patients (21). COVID-19 patients showed an imbalanced metabolism characterized by enhanced protein metabolism and suppressed carbohydrate-oriented catabolism, which might impel putrefactive gut microbiota dysbiosis in COVID-19 patients (19). This potentially led to the reinforcement of systemic inflammation. SARS-CoV-2-induced variations in intestinal microorganisms and metabolic capacities warrant further exploration. It is intriguing how SARS-CoV-2 affects microbial metabolism in the gut.

Remarkably, COVID-19 patients were detected positive for SARS-CoV-2 in the faeces during disease course or even after clearance of viral respiratory infection by viral RNA metagenomic sequencing (23). Such prolonged viral existence in patients without gastrointestinal symptoms underscored the possibility of intestinal infection and faecal-oral transmission of SARS-CoV-2. SARS-CoV-2-positive faecal samples were enriched in the opportunistic pathogens *Collinsella aerofaciens*, *Collinsella tanakaei*, *Morganella morganii* and *Streptococcus infantis*, while SARS-CoV-2-negative samples had an overgrowth of short-chain fatty acid (SCFA)-producing bacteria, *Alistipes onderdonkii*, *Bacteroides stercoris*, *Lachnospiraceae bacterium 1_1_57FAA* and *Parabacteroides merdae*. *A. onderdonkii* and *L. bacterium* were previously reported to play a protective role in ameliorating the severity of COVID-19 (31). These salutary bacteria might combat SARS-CoV-2 infection in the gut. Moreover, SARS-CoV-2-positive faecal samples presented higher functional capacity for amino acid biosynthesis, glycolysis and nucleotide *de novo* biosynthesis than those with low-to-none virus infectivity. These pathways might be essential for the survival, growth and metabolism of intestinal bacteria under the immunopathological state. Nevertheless, the impact of gut bacterial bioactivities on SARS-CoV-2 pathogenesis needs to be corroborated in future studies. The number of beneficial bacteria *Bacteroides uniformis* and *Parabacteroides distasonis* was increased in gut microbiome after viral clearance from faecal samples, whereas that of inflammation-related bacterium *Ruminococcus gnavus* was decreased. Inflammation-associated bacteria *Clostridium hathewayi*, *Enterococcus avium* and *R. gnavus* were enriched in patients who were constantly positive for SARS-CoV-2 in the faeces. COVID-19 patients exhibited significant alterations in gut microbiota composition with and without the presence of SARS-CoV-2 virus in the faeces, alluding to an ever-changing gut microbial landscape during disease course. Substantial research efforts are necessary to dissect the infectivity and pathogenesis of SARS-CoV-2 in the gut.

Currently, there is controversy concerning how long SARS-CoV-2-induced dysbiosis of gut microbiota will last. Gut microbiota profile of COVID-19 patients was characterized by an expansion of Prevotellaceae and a reduction of *Adlercreutzia*, Bacteroidaceae, *Eubacterium brachy*, *Faecalibacterium*, Lachnospiraceae and Ruminococcaceae compared with healthy and recovered subjects (24). Remarkably, recovered COVID-19 patients exhibited a descending trend in microbial diversity even after three-month recovery (25, 26). Recovered subjects had an enrichment of opportunistic pathogens (e.g., *Clostridium aldenense*, *Escherichia unclassified* and *Intestinibacter bartlettii*), inflammation-relevant pathogens (e.g., *Clostridium bolteae* and *Flavonifractor plautii*) and COVID-19-related bacteria *Clostridium ramosum* as well as a depletion of beneficial commensals including *Butyricicoccus pullicaecorum*, *Eubacterium hallii*, *F. prausnitzii*, *Fusicatenibacter saccharivorans*, *Intestinimonas butyriciproducens*, *Roseburia inulinivorans* and *Ruminococcus bromii*. Despite recovered patients had an insufficiency of SCFA-producing bacteria *E. hallii* and *Faecalibacterium*, the faecal levels of SCFAs in recovered patients was comparable to those in healthy controls, implying the supplementation from other SCFA-secreting bacteria. *Escherichia unclassified* and *I. bartlettii* were positively associated with persistent symptoms such as fatigue, myalgia and anorexia. *F. prausnitzii* and *I. butyriciproducens* negatively correlated with persistent symptoms (e.g., chest tightness and cough) after discharge. The underlying mechanisms through which gut microbiota influences the recovery of COVID-19 patients deserve in-depth investigation. A prospective study was previously carried out to longitudinally track alterations of gut microbiota composition in COVID-19 patients (44). The richness of gut microbiota was not restored to normal values even after six-month recovery. The significant loss of gut microbiota richness correlated with worse pulmonary functions. COVID-19 patients with attenuated postconvalescence richness presented higher levels of C-reactive protein (CRP) and disease severity during the acute phase, implying a strong association between intestinal dysbiosis and inflammatory responses in COVID-19. As stable ecosystems confer colonization resistance to opportunistic pathogenic microbes (45), constant attenuation of gut microbiota richness may have long-term biological impacts. A large population of COVID-19 patients suffer ongoing symptoms post initial recovery, termed long COVID-19 (46, 47). Persistent gut dysbiosis may contribute to long COVID-19, which needs to be experimentally verified. Disequilibrium of gut microbiota seems to correlate with the convalescence process of COVID-19, but this hypothesis needs further verification in larger cohorts.

Paradoxically, several studies indicated that gut microbiota could be restored with disease resolution. For instance, a longitudinal study showed that SARS-CoV-2 infection induced gut microbiota dysbiosis by reducing the relative abundance of Bacteroidetes and increasing the Firmicutes/Bacteroidetes ratio (48). However, these SARS-CoV-2-assocated variations in gut microbiota were reversed during the recovery phase. Nine recovered patients after at least two-week recovery exhibited a microbiota configuration similar to that of uninfected subjects (49). Gut microbiota richness increased in 31 recovered subjects after six-month recovery compared with patients with non-SARS-CoV-2 pneumonia (22). The relative abundance of Bacteroidetes was increased during SARS-CoV-2 infection and was declined after viral clearance. On the contrary, the number of Firmicutes was decreased in SARS-CoV-2-infected subjects and was elevated after recovery. SARS-CoV-2 positive patients had a surge of *Bacteroides* and a depletion of *Enterococcus*. The levels of *Blautia*, Lachnospiraceae and Ruminococcaceae were enriched after SARS-CoV-2 negativization, leading to the re-equilibrium of gut microbiota composition. Reportedly, *Bacteroides* had a positive correlation with the cytokine storm (39). *Blautia* possessed anti-inflammatory activities and was beneficial to the recovery from COVID-19 (50). These intestinal commensals might play an important role in disease resolution in COVID-19 patients. COVID-19 rehabilitation in turn facilitates the restoration of gut microbiota to the pre-infection state. It is speculative how gut microbiota affects the course of COVID-19, calling for subsequent studies in the future. The mechanisms responsible for the dynamic alterations in gut microbiota during SARS-CoV-2 infection warrant greater attention. Further studies are needed to define how long the imbalanced gut microbiota will last. Collectively, modifying gut microbiota may be instrumental in preventing the lasting impacts of SARS-CoV-2 infection and could improve the clinical outcome of SARS-CoV-2-infected subjects.

Gut mycobiota richness was also decreased in COVID-19 patients compared with healthy controls (27, 28). The number of fungi in the faeces of COVID-19 patients was markedly higher than in healthy controls. The association between increased fungal burden and susceptibility to SARS-CoV-2 infection deserves further verification. *Aspergillus*, *Candida parapsilosis*, *Malassezia yamatoensis*, *Moesziomyces aphidis*, *Mucor racemosus*, *Penicillium citrinum*, *Penicillium polonicum*, *Rhodotorula mucilaginosa*, *Talaromyces wortmannii*, *Trechispora* sp. and *Wallemia sebi* were depleted in COVID-19 patients. COVID-19 patients showed a predominance of *Candida albicans*. *Aspergillus niger* was positively associated with the incidence of diarrhoea. *P. citrinum* and *R. mucilaginosa* showed an inverse correlation with the levels of CRP and ACE. The diversity of mycobiota recovered at six months after recovery (28). The effects of gut mycobiota dysbiosis on COVID-19 pathogenesis and severity are worth exploring.

Altogether, SARS-CoV-2 infection exerts a prolonged adverse effect on gut microbiota composition. ACE2, an important receptor of SARS-CoV-2, can regulate amino acid malnutrition to affect gut microbiota and intestinal inflammation (51). Specifically, ACE2 affects the absorption of neutral amino acids, especially tryptophan, in the small intestine as a chaperone of amino acid transporters. ACE2 downregulation can inhibit the uptake of tryptophan, which reduces the antimicrobial peptide formation by Paneth cells, culminating in gut microbiota dysbiosis and gastrointestinal disorders related to intestinal microbiota imbalance (52). Previous reports showed that SARS-CoV-2 could decrease ACE2 expression in intestinal epithelial cells (53–55). It is thus inferred that SARS-CoV-2 infection contributes to gut microbiota dysfunction by downregulating ACE2. Nevertheless, this hypothesis remains to be validated in further studies. Diverse pathological variations including hypoxia and inflammation could be significant factors contributing to imbalanced gut microbiota (56). Moreover, the intake of antibiotics also causes gut microbiota dysbiosis. The use of unnecessary antibiotics should be avoided in COVID-19 treatment. More studies are required to achieve a deeper understanding of the exact mechanisms underlying dysregulated gut microbiota during COVID-19 pathogenesis. Dysbiotic gut microbiota can affect the response and susceptibility to viral infection in COVID-19 patients. Characterizing gut microbiota composition in COVID-19 patients can enrich our knowledge of the role of intestinal microorganisms in COVID-19 pathogenesis and lead to the identification of new therapeutic targets for COVID-19 treatment. The dynamic changes in gut microbiome during SARS-CoV-2 infection remain to be in-depth investigated. The exact effects of intestinal commensals on COVID-19 progression necessitate further study. Gut microbiota plays an important role in intestinal function and immunity. It is unknown whether the reduction of anti-inflammatory bacteria could give rise to chronic intestinal inflammation. Due to the persistence of abnormal gut microbiota, it is of great importance to continually monitor the intestinal health in recovered COVID-19 patients. Further clinical studies should be directed to clarify the long-term impact of SARS-CoV-2-induced gut microbiota dysbiosis on human health.

## 3 The antagonistic effects of commensal microbiota against SARS-CoV-2

**Figure 1:**
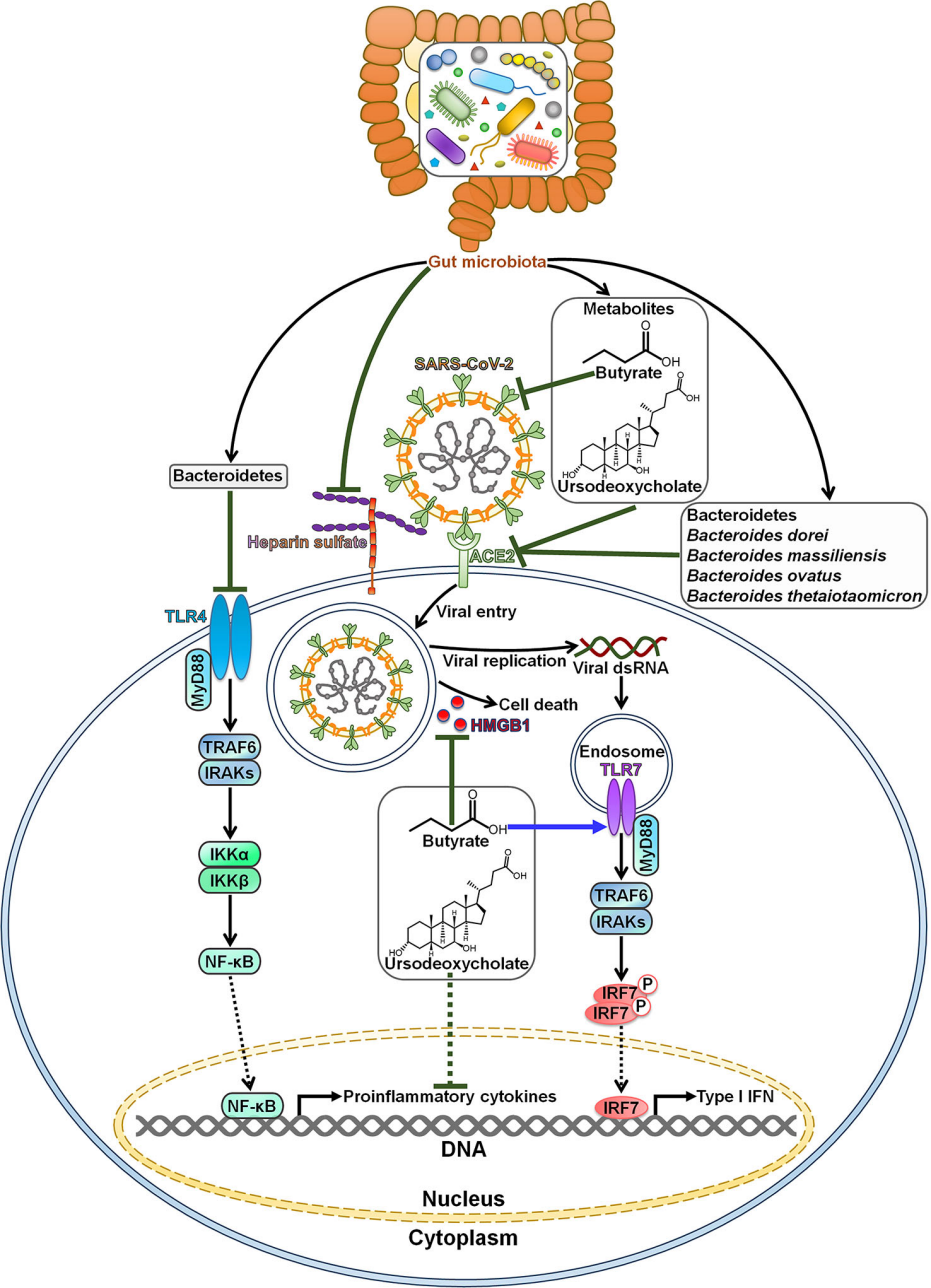
Roles of gut microbiota in resisting SARS-CoV-2 infection and pathogenesis. Bacteroidetes can block SARS-CoV-2-induced cytokine storm by targeting the TLR4 signaling pathway. Gut microbiota inhibits SARS-CoV-2 adhesion to target cells by regulating heparin sulfate. Gut microbiota-derived metabolite butyrate reduces the expression of membrane ACE2 and suppresses the activation of viral spike protein. Moreover, butyrate represses SARS-CoV-2-induced cell death by downregulating HMGB1. In addition, butyrate initiates antiviral immune responses by motivating the TLR7 signaling cascade. The metabolite ursodeoxycholate also exerts anti-SARS-CoV-2 effects. It prevents SARS-CoV-2 infection by blocking viral attachment to ACE2. Ursodeoxycholate has the ability to restrict the expression of pro-inflammatory cytokines, thereby ameliorating SARS-CoV-2-induced pathology. Species belonging to the Bacteroidetes phylum, such as *Bacteroides dorei*, *Bacteroides massiliensis*, *Bacteroides ovatus* and *Bacteroides thetaiotaomicron*, are found to restrain ACE2-mediated viral entry. TLR4, toll-like receptor 4; MyD88, myeloid differentiation factor 88; TRAF6, tumor necrosis factor receptor-associated factor 6; IRAKs, interleukin-1 receptor-associated kinases; IKKα, inhibitor of κB kinase α; IKKβ, inhibitor of κB kinase β; NF-κB, nuclear factor-κB; SARS-CoV-2, severe acute respiratory syndrome coronavirus 2; ACE2, angiotensin-converting enzyme 2; HMGB1, high mobility group protein 1; dsRNA, double-stranded RNA; TLR7, toll-like receptor 7; IRF7, interferon regulatory factor 7; IFN, interferon.

Gut microbiota is capable of impeding SARS-CoV-2 infection and pathogenesis (**Figure 1**). Commensal host bacteria interfered with SARS-CoV-2 adhesion to target cells by regulating heparin sulfate (57). Depletion of these microbes rendered individuals susceptible to SARS-CoV-2 infection. A sophisticated understanding of the effect of microbiota composition alterations on SARS-CoV-2 infection may be helpful in predicting susceptibility to viral infections and open up new opportunities for the treatment of COVID-19. COVID-19 patients had an enrichment of Bacteroidetes that was diminished after recovery (58). Bacteroidetes was reported to inhibit toll-like receptor 4 (TLR4) and ACE2-related signaling pathways (59, 60). The expansion of Bacteroidetes may constitute a component of host immune defense system, which remains to be further investigated. Furthermore, *Bacteroides dorei*, *Bacteroides massiliensis*, *Bacteroides ovatus* and *Bacteroides thetaiotaomicron*, known to reduce colonic ACE2 expression *in vivo*, showed negative correlation with faecal SARS-CoV-2 load in COVID-19 patients (31). These *Bacteroides* species might play a protective role in fighting SARS-CoV-2 infection by blocking ACE2-mediated viral entry.

Intestinal microbiota-derived metabolite butyrate could inhibit SARS-CoV-2 infectivity through downregulation of membrane ACE2 and inactivation of viral spike protein in gut epithelium (61). It also restricted SARS-CoV-2 replication by reducing the expression of high mobility group protein 1 (HMGB1). Butyrate could activate TLR signaling pathway. Butyrate might represent a preventive agent against SARS-CoV-2 infection. Nevertheless, the role of commensal-produced butyrate in opposing SARS-CoV-2 infection remains to be confirmed in preclinical and clinical studies. There was a negative relationship between intestinal *Collinsella* and COVID-19 mortality (62). *Collinsella*-derived ursodeoxycholate could prevent SARS-CoV-2 infection by blocking viral attachment to ACE2 and downregulating pro-inflammatory cytokines (interleukin-1β (IL-1β), IL-2, IL-4, IL-6 and tumor necrosis factor-α (TNF-α)) (63, 64). Ursodeoxycholate might relieve ARDS in COVID-19 by repressing cytokine storm syndrome (65). However, it will be necessary to ascertain whether this microbial product has beneficial effects against SARS-CoV-2 infection and COVID-19 development.

## 4 Effects of gut microbiota on SARS-CoV-2-related pathology

### 4.1 The role of gut microbiota in regulating susceptibility to SARS-CoV-2 infection

**Figure 2:**
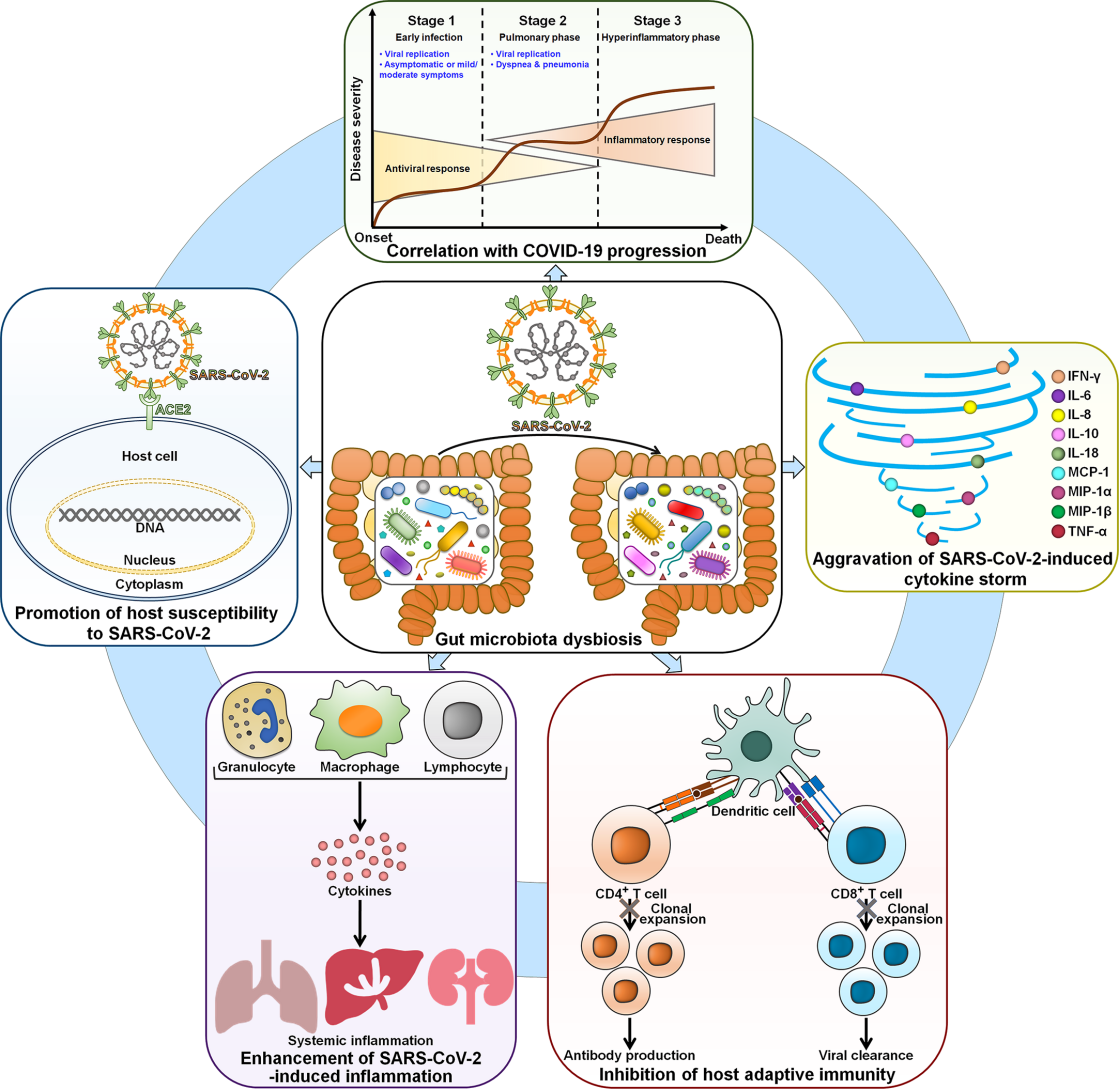
The involvement of dysbiotic gut microbiota in COVID-19 physiopathology. Gut microbiota affects the susceptibility of host cells to SARS-CoV-2 infection. SARS-CoV-2-induced gut microbiota perturbation contributes to COVID-19 deterioration. Thus, the gut microbiota signature is closely associated with clinical outcomes in COVID-19 patients. In terms of mechanism, gut microbiota exacerbates SARS-CoV-2-induced cytokine storm and leads to hyperinflammation. Moreover, gut microbiota is capable of abrogating host adaptive immune responses by inhibiting CD4^+^ T and CD8^+^ T cell expansion. SARS-CoV-2, severe acute respiratory syndrome coronavirus 2; ACE2, angiotensin-converting enzyme 2; IFN-γ, interferon-γ; IL-6, interleukin-6; IL-8, interleukin-8; IL-10, interleukin-10; IL-18, interleukin-18; MCP-1, monocyte chemoattractant protein-1; MIP-1α, macrophage inflammatory protein-1α; MIP-1β, macrophage inflammatory protein-1β; TNF-α, tumor necrosis factor-α.

It is proposed that gut microbiota contributes to the inter-individual variability in susceptibility to SARS-CoV-2 infection. The COVID-19 mortality and morbidity in the elderly are higher than in younger adults. The elderly gut microbiota was characterized by reduced microbial diversity, an enrichment of *Alistipes* and *Parabacteroides* and an underrepresentation of Firmicutes (66). Significant inter-individual differences in the amount of *Faecalibacterium* and *Ruminococcus* were observed among the elderly. These gut microbiota signatures may be an explanation for the high level of susceptibility to SARS-CoV-2 infection in this population. Gut microbiota could be a significant factor affecting individuals’ susceptibility to SARS-CoV-2 infection (**Figure 2**). However, there is a scarcity of direct evidence regarding the effect of gut microbiota composition on susceptibility to SARS-CoV-2 infection in healthy individuals. Further study is needed to compare the gut microbiota profiles among healthy individuals with different susceptibility toward SARS-CoV-2 infection. Gut microbiota dysbiosis may enhance the risk of SARS-CoV-2 infection by coordinating the expression of the viral entry receptor ACE2 in the gut (67). Supplementation of *Bifidobacterium longum* could elevate the expression of ACE2 receptor in mice, suggesting that altered *B. longum* might be a pivotal factor linked to COVID-19 susceptibility (68). The mechanisms employed by gut microbiota to regulate colonic ACE2 expression remain elusive and deserve further research. The use of broad-spectrum antibiotics could induce variations in gut microbiota, which may influence colonic ACE2 expression (69, 70). Therefore, substantial research efforts should be directed toward the interrelation between antibiotic-induced dysbiosis and COVID-19 infectivity. The colonic ACE2 expression was downregulated in germ-free (GF) rats co-housed with conventional rats compared with GF rats (70). This conventionalization caused enhanced systemic inflammation in conventionalized GF (GFC) rats, as evidenced by markedly increased levels of lipocalin 2 and neutrophilia. Metabolomic analysis demonstrated that the levels of hydroxy kynurenine, kynurenic acid and tryptophan metabolites were elevated in GFC rats, suggesting that altered expression of ACE2 might have an impact on intestinal microbiota metabolism. A better understanding of the reciprocal impact between gut microbiota and ACE2 expression in COVID-19-susceptible and -resistant patients will provide valuable insights into new treatment modalities for COVID-19. The intestinal microbe *Bacteroides* that was reduced in COVID-19 patients might impede viral invasion by regulating heparin sulfate, implying a potential interrelation between *Bacteroides* and susceptibility to SARS-CoV-2 infection (57). In addition, gut microbiota-derived signals have the ability to dominate immune cells for pro- and anti-inflammatory reactions, hence influencing the host’s susceptibility to SARS-CoV-2 infection (71). The association of indigenous microbes with susceptibility to SARS-CoV-2 infection is poorly understood. The mechanism through which gut microbiota affects COVID-19 susceptibility should be revealed in future studies.

### 4.2 The association between gut microbiota dysbiosis and COVID-19 severity

There is a significant correlation between gut microbiota complexity and COVID-19 severity (**Table 2**). SARS-CoV-2-driven gut dysbiosis may cause intestinal barrier disruption that contributes to the migration or passage of extra-intestinal bacteria (e.g., *Granulicatella* and *Rothia Mucilaginosa*) into the gut (30). Moreover, *Eubacterium dolichum* and *Prevotella copri* had a positive correlation with the faecal levels of SARS-CoV-2 while *Alistipes*, *Bifidobacterium*, *Clostridium citroniae*, *Dialister*, *Haemophilus*, *Haemophilus parainfluenzae*, *Ruminococcus* and *Streptococcus anginosus* showed an inverse association with the SARS-CoV-2 viral load. Antibiotic-naïve COVID-19 patients presented a higher abundance of opportunistic pathogens that could induce bacteremia such as *Actinomyces viscosus*, *Bacteroides nordii* and *C. hathewayi* than healthy controls and pneumonia controls (31). It was thus inferred that severe COVID-19 might be the outcome of secondary bacterial infection. Several members belonging to the Firmicutes phylum, *Coprobacillus*, *C. hathewayi* and *C. ramosum*, showed a positive correlation with COVID-19 severity (31). Reportedly, *Coprobacillus* was able to increase ACE2 expression in murine gut (60). *C. hathewayi* and *C. ramosum* were revealed to be connected with human infection and bacteremia (72, 73). In contrast, baseline faecal abundance of beneficial species *A. onderdonkii* and *F. prausnitzii* was inversely associated with COVID-19 severity (31). *Alistipes* species played a critical role in preserving intestinal immune homeostasis, while *F. prausnitzii* possessed anti-inflammatory abilities (74, 75). Nevertheless, substantial work remains to address the direct causality between gut microbiota dysbiosis and COVID-19 deterioration and prognosis.

The relative abundance of *Alistipes finegoldii*, *Clostridium innocuum* and *Ruthenibacterium lactatiformans* positively correlated with inflammatory biomarkers (e.g., CRP and WBC) and tended to increase with COVID-19 progression (32). Conversely, *Alistipes putredinis*, *Blautia luti*, *Dorea longicatena*, *F. prausnitzii* and *Gemmiger formicilis* were remarkably reduced in severe/critical patients and post COVID-19 patients. *Parabacteroides* was enriched while *F. prausnitzii* was depleted in patients with complications (e.g., hemodialysis). These intestinal microorganisms may be involved in the occurrence of complications in COVID-19. COVID-19 severity was correlated with increased abundance of *Bacteroides* and reduced abundance of *Bifidobacterium* and *Roseburium* (33). The immunoregulatory *Bifidobacterium* appeared to play a role in inhibiting SARS-CoV-2-induced cytokine storm. The loss of *Bifidobacterium* may be predisposing factor for severe disease. Additional studies are required to confirm and expand this assumption. *Aspergillus flavus*, *C. albicans* and *Candida auris* were strikingly enriched in COVID-19 patients but could not be detected in healthy controls (34). The expansion of pathogenic fungi might lead to gut mycobiota imbalance in COVID-19 patients. The persistent existence of these fungi probably caused a long-term detrimental effect on human health. Reportedly, *C. albicans* colonization exacerbated inflammation in the gut and extra-gut tissues (76). *Aspergillus* infection was capable of inducing pulmonary and respiratory manifestations (77). Thus, cough was prevalent in COVID-19 patients with an enrichment of *Aspergillus* species, implying *Aspergillus*-induced systemic effect and the perplexing connection between the gastrointestinal and respiratory systems. Consistently, the abundance of *Aspergillus* species seemed to be positively associated with disease severity (34). The variation in gut microbiota composition might be attributable to SARS-CoV-2 infection. *C. albicans* and *Aspergillus* species potentially resulted in disease progression in COVID-19 patients. These mutual effects are largely unknown and need more detailed investigation.

### 4.3 Gut microbiota-mediated immune dysregulation in COVID-19

Gut microbiota performs a critical role in the development and functionality of host immune system (35). It is thus supposed that gut microbiota could affect COVID-19 severity through actions on the host immune system (**Figure 2**).

#### 4.3.1 The reciprocal impacts between gut microbiota and the immune system

Gut microbiota is implicated in host inflammation and disease progression in COVID-19. Commensal and pathogenic microbes act as a vital source of microbe-associated molecular patterns (MAMPs) and pathogen-associated molecular patterns (PAMPs) (78). SARS-CoV-2-induced direct damage of enterocytes and gut microbiota dysbiosis could enhance intestinal permeability, facilitating the translocation of microbe-derived signals into the circulating system (79). Pattern recognition receptors (PRRs), such as TLRs, can sense these microbial factors and initiate distinct immunologic reactions depending on the type of cell, ligand or receptor. Due to the breach of intestinal barrier, opportunistic pathogens could enter the circulation and result in systemic inflammation (80). Thus, gut barrier dysfunction interrupts the critical equilibrium between intestinal microbes and host immunity in COVID-19.

SARS-CoV-2 can translocate from the lungs to the gastrointestinal tract (81). Once SARS-CoV-2 reaches intestinal epithelial cells, the virus invades the enterocytes *via* its receptor ACE2. After numerous viruses are secreted from epithelial cells, innate immune cells (e.g., dendritic cells and macrophages) then detect and bind viral PAMPs, and this eventually results in the excessive release of proinflammatory cytokines. The inflammatory cascade further drives gut microbiota dysbiosis (82). Host immune response should be effective at antagonizing SARS-CoV-2 infection. In some circumstances, an over-reactive immune reaction may occur, resulting in extensive lung or multiorgan damage (83). Reportedly, the levels of various chemokines and cytokines, such as basic fibroblast growth factor (FGF), granulocyte colony-stimulating factor (G-CSF), granulocyte-macrophage colony-stimulating factor (GM-CSF), monocyte chemoattractant protein-1 (MCP-1), vascular endothelial growth factor (VEGF), IL-2, IL-7, IL-10 and TNF-α were higher in COVID-19 patients than healthy controls (84). Particularly, the levels of some factors (e.g., G-CSF, MCP-1, IL-2, IL-7, IL-10 and TNF-α) were increased in severe patients relative to nonsevere patients. An integrative analysis of multi-omics data from patients with gastrointestinal manifestations revealed that *Blautia*, *Lactobacillus* and *Ruminococcus* positively correlated with proinflammatory cytokines, including interferon-γ (IFN-γ), IL-2, IL-4, IL-6, IL-8, IL-10 and TNF-α (35). In contrast, *Bacteroides*, Clostridiales and *Streptococcus* were inversely associated with these proinflammatory cytokines. Gut microbiota composition of COVID-19 patients was found to be connected with plasma concentrations of aspartate aminotransferase (AST), C-X-C motif ligand 10 (CXCL10), CRP and lactate dehydrogenase (LDH) (39). COVID-19-depleted *Bifidobacterium adolescentis*, *Eubacterium rectale* and *F. prausnitzii* could regulate the gastrointestinal immune response (85–87). It is thus conceivable that the reduction of these microorganisms could disrupt the intestinal immune homeostasis and lead to exuberant inflammation. Furthermore, correlative blood samples indicated an interrelation between gut microbiota dysbiosis, multiplication of inflammatory mediators and severity of systemic inflammation in COVID-19 (39).

Gut microbiota plays an important role in the evocation and training of the host immune system (88). Immunoregulatory signals and metabolites, such as SCFAs and bile acids, produced by intestinal commensals (e.g., *Bacteroides*, *Bifidobacteria* and *Lactobacillus*) can bind to the corresponding receptors on innate immune cells, thus regulating their metabolism and functions (89). The balance between proinflammatory cells (e.g., T helper 17 (Th17)) and inflammatory regulatory cells (regulatory T cells (Tregs)) is essential for intestinal health and homeostasis, which is ultimately controlled by intestinal microorganisms (78). Gut microbiota dysbiosis is a contributing factor to immune dysfunction. For instance, SARS-CoV-2-induced overgrowth of opportunistic pathogens including *Burkholderia contaminans* and *Enterococcus faecalis* could contribute to the suppression of T cell immunity (36, 37). Impaired immune responses could lead to an increased susceptibility to pathogen infection, which fosters the perturbation in intestinal flora. Collectively, there is a complex interplay between gut microbiota and systemic immunity in COVID-19. However, further research in this emerging field is warranted.

#### 4.3.2 Gut microbiota-induced inflammation and cytokine storm

Gut dysbiosis can disturb the homeostatic relationship between resident microbiota and intestinal immune system, leading to chronic inflammatory diseases (90). COVID-19 microbiota was characterized by a reduction of anti-inflammatory bacteria including *Bacteroides plebeius*, *F. prausnitzii*, *Lachnospira*, *Prevetolla* and *Roseburia* (38). Decreased levels of *Lachnospira*, *Prevetolla* and *Roseburia* were associated with enhancement of inflammatory responses (91). Consistently, the level of the inflammatory cytokine IL-21 was elevated in COVID-19 patients. Gut microbiota dysbiosis might be linked to the exacerbation of inflammatory responses in COVID-19. The loss of butyrate-producing anti-inflammatory bacteria (e.g., *Faecalibacterium* and *Roseburia*) as well as the upregulation of proinflammatory mediators (e.g., CRP, IL-6 and soluble IL-2 receptor (sIL2R)) was more pronounced in severe/critical COVID-19 patients than mild patients (92). The depletion of anti-inflammatory bacteria is likely to lead to excessive inflammation in severe/critical patients. The correlation between gut microbiota and inflammatory factors has been preliminarily established. For instance, two COVID-19-enriched bacteria *Akkermansia muciniphila* and *B. dorei* exhibited a positive relationship with IL-6, CXCL8 and IL-1β (39). Conversely, six depleted gut commensals in COVID-19 including *B. adolescentis*, *C. aerofaciens*, *Coprococcus comes*, *D. longicatena*, *E. rectale* and *F. prausnitzii* were negatively connected with CXCL10. *C. aerofaciens*, *C. comes*, *D. formicigenerans*, *D. longicatena* and *Ruminoccocus obeum* negatively correlated with IL-10. *C. aerofaciens* and *C. comes* were negatively associated with TNF-α, and *C. comes* and *E. rectale* inversely correlated with C-C motif chemokine ligand 2 (CCL2) (39). The relative abundance of COVID-19-depleted Clostridia showed an inverse association with the IFN-γ level, while the count of COVID-19-enriched Actinobacteria was positively connected with the glycoprotein 130 (gp130)/soluble IL-6 receptor subunit β (sIL-6Rb) level (40). Therefore, intestinal flora disturbance might lead to microbial-mediated immune dysregulation (**Figure 2**). Future research efforts should be made to verify the causal relationship between gut microbiota and excessive inflammation in COVID-19 patients.

The impairment of intestinal barrier integrity is deemed as critical inducer of systemic inflammation in COVID-19. The existence of intestinal microbiota in the plasma as well as increased plasma levels of gut permeability markers fatty acid binding protein 2 (FABP2), lipopolysaccharides (LPS) and peptidoglycan (PGN) indicated the intestinal barrier dysfunction in COVID-19 (93). Severe COVID-19 was associated with higher levels of intestinal barrier integrity marker (zonulin) and microbial translocation markers (β-glucan and lipopolysaccharide binding protein (LBP)) (94). Zonulin, β-glucan and LBP positively correlated with the factors of systemic inflammation and immune activation including CRP, IL-6 and IL-10. Enhancement of tight junction permeability and microbial translocation could contribute to microbiota-mediated myeloid inflammation. As expected, the levels of monocyte and neutrophil inflammation markers (soluble CD14 (sCD14) and myeloperoxidase (MPO)) were increased in the severe group compared with mild and control groups, alluding to the association between microbial translocation and COVID-19 severity. Citrulline, a marker of intestinal function, was remarkably decreased in severe COVID-19, while the marker of intestinal dysbiosis succinic acid showed an opposite trend (94). The kynurenine/tryptophan ratio was increased in severe patients. The citrulline level was inversely associated with IL-6, whereas the level of succinic acid and the kynurenine/tryptophan ratio were positively correlated with the concentration of IL-6. It was plausible to assume that disrupted gut functions concomitant with dysregulated metabolic activities could be potential forces contributing to COVID-19-associated inflammation. The loss of gut barrier integrity in severe COVID-19 may be attributed to dysregulated gut microbiota. Reportedly, COVID-19-enriched *Eggerthella* could enhance intestinal permeability, which might allow the transfer of intestinal microbes and toxins to the circulation and exacerbate inflammation-induced injuries (40). It will be important to adequately reveal the mechanisms underlying intestinal barrier impairment. The potential effect of microbial translocation on mucosal and systemic immune responses must be a research priority. In addition, intestinal microbe-produced metabolites may act as regulators of the inflammatory response in COVID-19. COVID-19 patients exhibited reduced capacity for SCFA production and L-isoleucine biosynthesis due to the loss of *F. prausnitzii* (95). Impaired SCFA and L-isoleucine biosynthesis showed an intimate association with elevated plasma levels of CRP and CXCL10 (96, 97). The inverse association between these microbial products and proinflammatory cytokines underscored their significance in COVID-19 pathology and disease outcome. Collectively, the linkage between altered microbial functions and COVID-19 severity opens new horizons for dissecting the role of gut microbiota in COVID-19 development. The long-term implications of disrupted metabolic pathways during COVID-19 should be an important subject of future studies.

Increasing evidence has indicated the bidirectional crosstalk between gut microbiota and lung, called the gut-lung axis (78). On the one side, the gut can affect the progression of lung disorders. Under pathologic conditions, gut microbiota dysbiosis damages intestinal barrier integrity, which enables microbial components and metabolites to translocate into the lung tissues *via* the bloodstream. This effect can lead to sepsis and even ARDS (98). On the other side, pulmonary inflammation (e.g., respiratory viral infections) induces the perturbation of gut microbiota. It is reasonable to propose that the bidirectional gut-lung crosstalk has a pivotal role in SARS-CoV-2 pathogenesis and disease severity in COVID-19 patients. The gut microbiota in COVID-19 is characterized by a remarkable depletion of beneficial species and an enrichment of opportunistic pathogens. These variations eventually contribute to intestinal disruption, migration of pathogenic microorganisms across the intestinal mucosa, secondary microbial infections, increased inflammatory responses, multiorgan failure and unsatisfactory clinical outcome (99). SARS-CoV-2-induced gut dysbiosis may be a fundamental pathophysiological process contributing to COVID-19-associated hyperinflammation. The role of gut microbiota in mediating the interaction between SARS-CoV-2 and host innate immunity should be explicitly defined in future studies.

Given the linkage between gut microbiota and proinflammatory cytokines, the cytokine storm may result from SARS-CoV-2-dependent shifts in gut microbiota composition (**Figure 2**). The faecal level of IL-18, a proinflammatory cytokine produced by intestinal cells, was higher in COVID-19 patients than in healthy controls (41). Faecal IL-18 levels showed a positive correlation with the abundance of *Citrobacter*, *Fusobacterium* and *Peptostreptococcus*. Intestinal epitopes derived from *E. faecalis* GroEL were enriched in COVID-19 patients with fever and showed a positive relationship with IL-6 and IL-10 (37). In contrast, the anti-inflammatory bacterium *Eubacterium ramulus* that was overabundant in patients with non-fever was negatively linked to IL-6. SARS-CoV-2-induced intestinal dysbiosis resulted in the alterations in gut metabolites including amino acids, carbohydrates and neurotransmitters (100). Microbe-mediated amino acids were positively associated with increased levels of inflammatory cytokines (CXCL9, CXCL10, IFN-γ and IL-6) and negatively correlated with decreased levels of cytokines (IL-9 and IL-17) in COVID-19. COVID-19-depleted carbohydrate metabolites and neurotransmitters had a positive correlation with reduced levels of cytokines (e.g., CCL22, IL-12 and IL-13) while negatively correlated with increased levels of inflammatory cytokines (e.g., IL-6 and IL-10). Thus, gut metabolites might be implicated in cytokine dynamics in COVID-19. The contribution of gut microbiota and its derived metabolites to COVID-19 development should be corroborated in *in vivo* experimental models. Disrupted gut barrier favored the translocation of intact microbes or microbial components (e.g., LPS) into the systemic circulation, which impelled the secretion of proinflammatory cytokines, resulting in the exacerbation of cytokine storm in COVID-19 patients (93, 101). As expected, the plasma concentrations of proinflammatory cytokines IFN-γ, IL-6, IL-8, MCP-1, macrophage inflammatory protein (MIP)-1α, MIP-1β and TNF-α were elevated in COVID-19 subjects (93). The mechanisms by which gut microbiota modulates the cytokine response in COVID-19 are still equivocal and should be a subject of future studies.

#### 4.3.3 The interaction between gut microbiota and adaptive immunity

Mild/moderate COVID-19-enriched *Blautia obeum*, *Coprococcus catus* and *C. comes* and severe/critical COVID-19-depleted *Roseburia intestinalis* showed a positive relationship with the number of lymphocytes, CD3^+^ T cells, CD4^+^ T cells and CD8^+^ T cells and lymphocyte proportion (42). Altered gut microbiota led to inhibition of superpathways of polyamine biosynthesis II and sulfur oxidation in severe/critical COVID-19. The suppression of polyamine biosynthesis may contribute to reduced T cell proliferation and uncontrolled production of cytokines, which necessitates further verification. Further metabolomics studies are required to comprehensively delineate the crosstalk among gut microbiota, microbial metabolites and host adaptive immune system. The opportunistic pathogen *E. faecalis* that was overabundant in COVID-19 patients had a negative correlation with CD8^+^ T cells (37). COVID-19-enriched *B. contaminans* was negatively associated with the levels of lymphocytes, CD3^+^ T cells and CD4^+^ T cells (36). The microbial metabolic activity (e.g., glucose metabolism) might mediate the effect of gut microbiota dysbiosis on immune reaction in COVID-19. The circulating level of the microbial translocation marker LBP, which was markedly elevated in severe patients, had a correlation with lymphocytes. It is thus inferred that the impairment of gut barrier function may mediate the crosstalk between gut microbiota and T cell immunity in COVID-19. The effects of gut microbiota on adaptive immunity during COVID-19 development are worthy of greater attention.

### 4.4 Gut microbiota as potential therapeutic targets for COVID-19

A growing body of evidence has suggested the dynamic variation of gut microbiota composition during SARS-CoV-2 infection. It was reported that COVID-19 patients had an expansion of *Streptococcus* and a contraction of Ruminococcaceae, *Agathobacter*, *Anaerostipes*, *E. hallii*, *Fusicatenibacter*, unclassified Lachnospiraceae and *Roseburia* when compared to healthy controls (102). Compared with non-COVID-19 subjects, COVID-19 patients had a depletion of *Bariatricus comes*, *Blautia_A obeum*, *Blautia_A wexlerae*, *D. formicigenerans*, *F. prausnitzii_D*, *F. saccharivorans* and *Faecalibacterium sp900539945* (103). Importantly, COVID-19 patients at different severity stages (mild, moderate and severe) show varied gut microbiota signatures. A previous study showed that moderate and severe COVID-19 patients presented a lower Firmicutes/Bacteroidetes ratio, a higher abundance of Proteobacteria and a lower proportion of beneficial bacteria including *Lachnospira* and *Roseburia* than mild patients (104). Severe patients has an overrepresentation of *Actinomyces naeslundii* and *S. salivarius*, while recovered patients had a high abundance of *B. luti* and *C. comes* (105). *Agathobacter rectalis*, *Alistipes senegalensis*, *Bacteroides finegoldii*, *Bacteroides xylanisolvens*, *B. adolescentis*, *E. coli*, *Gemmiger formicillis*, *Pantoea agglomerans*, *Rodentibacter trehalosifermentans* and *Streptococcus parasanguinis* were only present in mild patients. Moreover, the microbiota signatures were correlated with common symptoms in COVID-19, such as dry cough, dyspnea, fever and headache. Manipulation of gut microbiota may be an auxiliary treatment option to mitigate the clinical presentations in COVID-19 patients. However, gut microbiota signatures in COVID-19 patients await further verification in larger cohorts. Longitudinal studies are needed to explore the changes in gut microbiota composition at distinct disease stages in COVID-19 patients.

The relative abundance of probiotic bacteria (*Bifidobacterium* and *Lactobacillus*) and butyrate-producing bacteria (*Clostridium butyricum, Clostridium leptum*, *E. rectale* and *F. prausnitzii*) markedly diminished as disease severity increased (106). The number of conditional pathogenic bacteria *Enterococcus* and Enterobacteriaceae was elevated with COVID-19 severity. Therefore, gut microbiota composition may reflect disease severity in COVID-19 patients. The perturbation of gut microbiota may increase the risk of mortality in COVID-19 patients. Increased abundance of Proteobacteria and decreased levels of secondary bile acids and desaminotyrosine were found to correlate with mortality in critical COVID-19 patients (107). These microbial metabolites were also connected with the occurrence of respiratory failure resulting in mechanical ventilation. The genera *Bacteroides* and *Parabacteroides* were decreased in non-survivor patients with COVID-19 when compared to survivors (108). Hence, further study of intestinal microbiota relevant to better clinical outcomes will facilitate the development of effective therapeutic options for COVID-19. Although several registered clinical trials involving regulation of gut microbiota for COVID-19 management are in progress (109), more clinical evidence is still required to substantiate the safety and efficacy of microbiota-directed therapies.

## 5 Potential therapeutic role of microbiota-targeted treatments in COVID-19

### 5.1 Faecal microbiota transplantation

**Figure 3:**
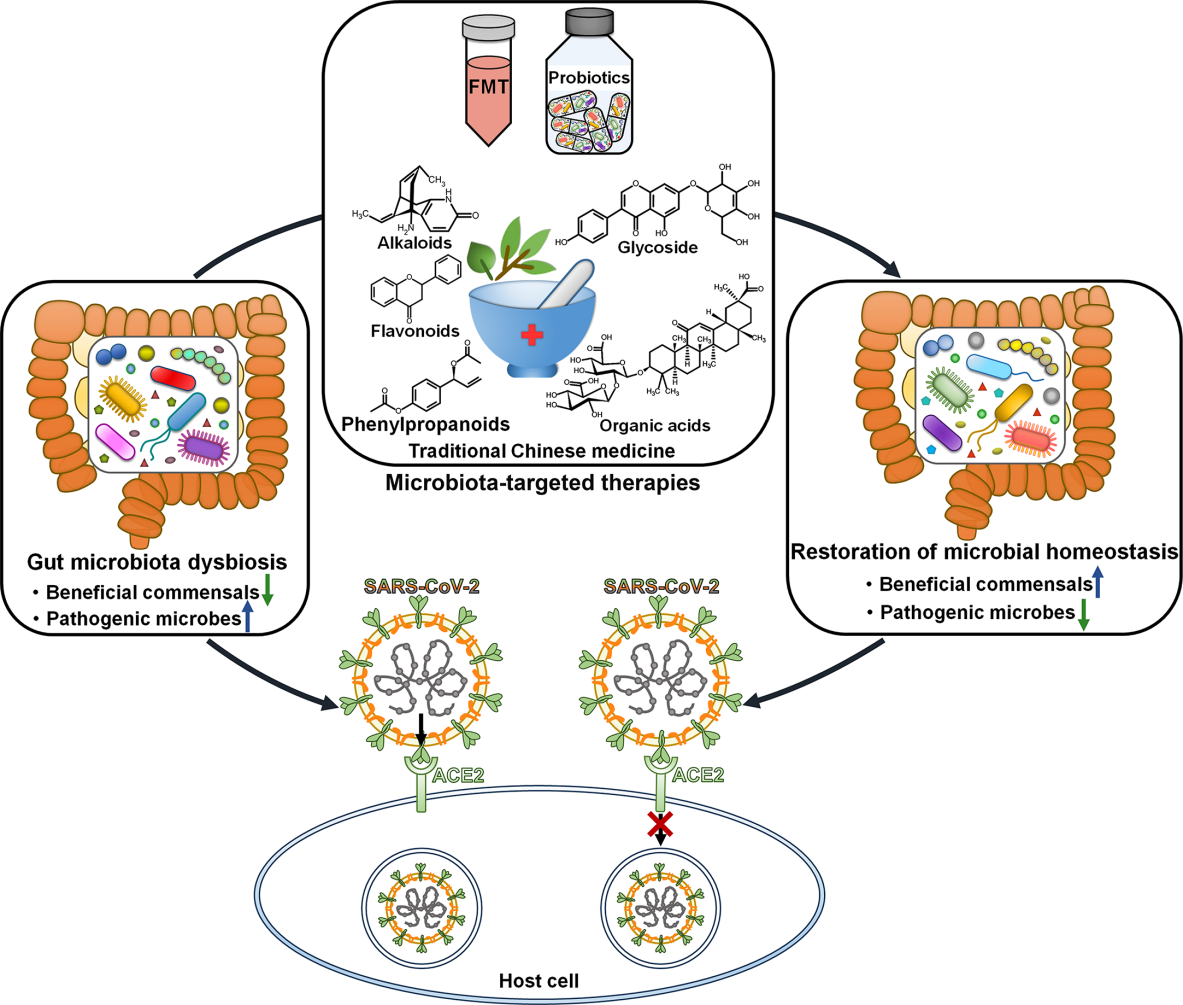
Therapeutic potential of microbiota-directed treatments in COVID-19. Microbiota-targeted therapies, including FMT, probiotics and traditional Chinese medicine, may reverse gut microbiota dysbiosis and play a protective role in antagonizing SARS-CoV-2 infection and pathogenesis. FMT, faecal microbiota transplantation; SARS-CoV-2, severe acute respiratory syndrome coronavirus 2; ACE2, angiotensin-converting enzyme 2.

Faecal microbiota transplantation (FMT) from healthy donors could mitigate gastrointestinal symptoms in discharged COVID-19 patients (110). The richness of gut microbiota was significantly increased in COVID-19 patients after FMT. Moreover, FMT treatment partially ameliorated gut dysbiosis by ascending the level of Actinobacteria, *Bifidobacterium* and *Faecalibacterium* and descending the level of Proteobacteria. Manipulating gut microbiota *via* FMT may represent a promising therapeutic intervention for COVID-19 (**Figure 3**). Gut microbiota dysbiosis in discharged COVID-19 patients awaits further validation through large-scale studies. Sustained efforts should be dedicated to understanding the impact of altered gut microbiota on post-infection recovery.

### 5.2 Bacteriotherapy

A retrospective, observational cohort study that included 200 patients with severe COVID-19 were conducted to assess the efficacy of oral bacteriotherapy composed of *Bifidobacterium lactis* DSM 32246, *B. lactis* DSM 32247, *Lactobacillus acidophilus* DSM 32241, *Lactobacillus brevis* DSM 27961, *Lactobacillus helveticus* DSM 32242, *Lactobacillus paracasei* DSM 32243, *Lactobacillus plantarum* DSM 32244 and *Streptococcus thermophilus* DSM 32245 (111). It turned out that the best available therapy (BAT) plus oral bacteriotherapy could effectively prevent disease progression in COVID-19 patients. Moreover, the mortality was lower in patients treated with BAT plus oral bacteriotherapy than those who only received BAT. Further analysis showed that oral bacteriotherapy served as an independent parameter related to a decreased risk for death. Larger prospective clinical trials are still necessary to verity the aforementioned results. Twelve COVID-19 patients were treated with probiotics that included *B. lactis* subsp. HNO19, *Lactobacillus casei* subsp. Lc-11, *L. plantarum* subsp. Lp-15, *B. lactis* subsp. B420, *B. longum* subsp. BL05, *Lactobacillus format* subsp. Lg-36, *Lactobacillus rhamnosus* subsp. Lr-32, *L. paracasei* subsp. Lpc-37, and *Lactobacillus salivariu* (20). The use of probiotics could partially reverse COVID-19-related gut microbiota dysbiosis. The dominant bacteria shifted from *Escherichia-Shigella* to *Bacteroides*, *Enterococcus* and *Veillonella* after treatment. The probiotics group had a reduction of *Rhodococcus* and conditional pathogenic bacteria *E. coli* and *K. pneumonia* and an enrichment of *Clostridium XlVa*, *F. prausnitzii* and *R. hominis*. The gut microbiota profiles may be varied among different COVID-19 patients or during the courses of probiotic-based treatment, calling for continual research efforts to comprehensively reveal the characteristics of gut microbiota in COVID-19. A total of 375 COVID-19 patients were enrolled in a retrospective clinical trial to investigate the therapeutic efficacy of a probiotic combination that included *Bifidobacterium*, *Enterococcus* and *Lactobacillus* (112). Of these, 179 patients were treated with standard care plus probiotics. The administration of probiotics was correlated with improved clinical outcomes in COVID-19 patients, as evidenced by dramatic reductions in duration of fever, viral shedding and hospital stay. These findings may open new avenues toward the development of innovative COVID-19 therapies. It is worth mentioning that more randomized controlled trials are necessary to verify the clinical benefit of probiotics in treating COVID-19.

A randomized, quadruple-blinded, placebo-controlled trial was conducted to assess the therapeutic potential of a probiotic formula that included *Lactiplantibacillus plantarum* strains KABP022, KABP023 and KABP033 and *Pediococcus acidilactici* strain KABP021 in 300 COVID-19 patients (113). This probiotic formula was well-tolerated and reduced nasopharyngeal viral load, lung infiltrates as well as duration of clinical symptoms (e.g., diarrhoea, dyspnea, headache and myalgia) compared with placebo. Moreover, the probiotic group had a higher serum level of virus-specific IgG and IgM than the placebo group. Probiotic treatment had no significant effect on gut microbiota composition. It was proposed that this probiotic formula might act on the gut-lung axis *via* interaction with host immune system. More clinical studies are warranted to testify these findings and uncover the mechanism of action of probiotic. A formula of *Bifidobacteria* strains, galactooligosaccharides, resistant dextrin and xylooligosaccharide (SIM01) efficiently diminished nasopharyngeal SARS-CoV-2 viral load and fostered the generation of SARS-CoV-2 antibody in COVID-19 patients (114). SIM01 formula also reduced the plasma levels of proinflammatory markers IL-1 receptor antagonist (IL-1RA), IL-6, MCP-1, macrophage colony-stimulating factor (M-CSF) and TNF-α. It also restored gut microbiota dysbiosis in COVID-19 patients by increasing commensal bacteria (e.g., *Bifidobacterium* spp., *Eubacterium* spp. and *F. prausnitzii*) and decreasing opportunistic pathogens (e.g., *Bacteroides* spp. and *E. coli*). Thus, manipulation of gut microbiota can be beneficial to strengthen host immune responses against COVID-19.

### 5.3 Traditional Chinese medicine

Compelling evidence has demonstrated that traditional Chinese medicine (TCM) can help to alleviate clinical symptoms and improve prognosis in COVID-19 patients (115). For instance, Huashi Baidu granules, Lianhua Qingwen granules and Qingfei Paidu decoction were reported to relieve the main clinical presentations of cough, chest tightness, fatigue and fever (116–118). Particularly, Lianhua Qingwen granules, Qingfei Paidu decoction and Shuanghuanglian oral liquid facilitated the absorption of pulmonary inflammation and reduced nucleic acid negative time and the average length of hospitalization (119–121). Furthermore, TCM can inhibit the inflammatory response and enhance antiviral immune function. Reportedly, Xuanfei Baidu decoction reduced the levels of CRP and erythrocyte sedimentation rate (ESR) and increased lymphocyte amount (122). Maxing Shigan decoction diminished the levels of alanine aminotransferase (ALT), AST, CRP and IL-6 and elevated the counts of CD4^+^ and CD8^+^ T cells (123). Tanreqing capsule elevated the number of CD3^+^ T cells (124).

Gut microbiota in COVID-19 patients is disordered, leading to pathogen infection and disease deterioration (115). Gut microbiota can adjust the host immune system and has emerged as a turning point of the prognosis of COVID-19 patients. TCM may be effective in treating COVID-19 by correcting gut microbiota imbalances and improving the host immune reactions. Most TCM is taken orally, enabling TCM to interact with numerous microbes in the intestine. Gut microbiota plays a vital role in the pharmacological effects of TCM by affecting the metabolic transformation and bioavailability of TCM. TCM in turn alters gut microbiota composition by favoring the growth of beneficial microbes and suppressing the reproduction of harmful species, contributing to the preservation of a healthy intestinal environment and the improvement of body immunity (125). The interplay between the effective components of TCM and gut microbiota has been investigated. Alkaloids, flavonoids, glycoside, organic acids and phenylpropanoids are the primary categories of TCM ingredients that have the ability to alter gut microbiota (126). Alkaloids are a class of nitrogen-containing organic compounds and possess important physiological functions (127). The structural features of alkaloids are commonly small molecules, ether bonds and coordination bonds, which may undergo hydrolysis and dehydration reactions under the action of intestinal microorganisms (128). The majority of flavonoids (except flavanols) naturally bind to sugars to form β-glycosides, thus they are not readily absorbed by the small intestine (129). Most glycosylated flavonoids reach the intestine, where intestinal microorganisms can metabolize the flavonoids to generate phenolic acids or other metabolites. Flavonoids exert a modulatory effect on gut microbiota, while intestinal commensals can regulate flavonoid activity and bioavailability (130). Intestinal microbes produce esterases, glycoside hydrolases and lyases to break down sugar chains to produce energy (131). The important end products generated by polyglycolysis are SCFAs that include acetic acid, butyric acid and propionic acid (132). TCM may enhance the population of SCFA-producing bacteria (e.g., anaerobic bacteria and *Bifidobacterium*) to strengthen the barrier function of the intestine and restrict the inflammatory response of the intestinal mucosa (133). Intestinal microbes secrete esterase to hydrolyze organic acids in the intestine. Particularly, lactic acid, an organic acid produced by anaerobic glucose metabolism, is capable of suppressing the growth of pathogenic bacteria. Moreover, specific microbes (e.g., *Clostridium*, *E. coli* and *Lactobacillus*) can convert carbohydrates or polyphenols into organic acids (134). Due to a lactone structure, phenylpropanoids are prone to demethylation and lactone hydrolysis by intestinal microbiota (135). Future work is necessary to clarify the interaction between TCM and gut microbiota, which will provide new ideas and new targets for the management of COVID-19. It is conceivable that TCM could alleviate COVID-19-associated gut microbiota dysbiosis, hence ameliorating the pathological conditions in patients. However, the mechanisms underlying TCM-mediated regulation of gut microbiota need to be further revealed.

## 6 Conclusions and future perspectives

Gut microbiota exhibits dynamic alterations during the course of COVID-19. The exact effect of SARS-CoV-2 infection on gut microbiota composition should be determined. Since therapeutic drugs could cause the variations in gut microbiota composition, it is essential to explore the gut microbiota configuration in treatment-naïve patients. The underlying mechanism of gut microbiota dysbiosis remains largely unknown. Continued research efforts are required to ascertain whether the enrichment of specific gut microorganisms is delicately coordinated or occasionally occurs owing to the decrement of other bacterial commensals. It is conjectured that SARS-CoV-2 affects gut microbiota composition *via* direct and indirect manners. SARS-CoV-2 may interrupt gut microbiota homeostasis through downregulation of ACE2. SARS-CoV-2-induced inflammation also leads to gut microbiota dysbiosis. Follow-up studies are required to elucidate the mechanisms exploited by SARS-CoV-2 to orchestrate the host gut microbiota. The duration of altered gut microbiota remains to be determined in further studies. An in-depth investigation on gut microbiome in convalescent patients would be conducive to unearthing suitable nutritional interventions for COVID-19 patients during the recovery phase. Furthermore, it is critical to address whether gut microbiota dysbiosis predisposes recovered subjects to future health problems.

The gut microbiome signatures serve as prospective biomarkers for risk assessment and prognostic prediction in COVID-19 patients. However, different treatments can affect gut microbiota configuration. Properly controlled studies including different severity groups of COVID-19 patients with minimal variations across clinical managements are needed to verify the clinical value of gut commensals. Gut microbiota disturbances may be a contributor to COVID-19 progression. Nevertheless, the causative linkage between gut dysbiosis and disease severity in COVID-19 necessitates additional studies. The perplexing role of gut microbiota in COVID-19 progression has not been fully understood yet. Given the association of the cytokine profile with hyperinflammation in COVID-19, intensive effort should be directed to characterize the interplay between gut microbiota and host cytokine responses. It is still intriguing which bacterial species are involved in COVID-19-associated pathologies. Some microorganisms induce protective immune response against SARS-CoV-2 infection, while pathogenic bacteria act to antagonize host defensive machineries. The overall impact of gut microbiota on host antiviral immunity is worthy of in-depth investigation. Owing to intestinal barrier impairment, commensal microbes and their metabolites can enter the circulation and augment inflammation-induced damages. Great attention must be concentrated on the complex intercourse between gut microbiota and host immune system during COVID-19 pathogenesis. Given the close relationship between gut microbiota and COVID-19 physiopathology, manipulation of gut microbiota may represent an alternative strategy for COVID-19 treatment. Preliminary clinical studies have proven the therapeutic efficacy of FMT, live biotherapeutic products and probiotics in COVID-19 treatment. Since the majority of these studies are retrospective, it should be cautious to draw the final conclusion. Further preclinical and clinical studies are urgently needed to reveal the mechanistic links underlying the therapeutic potential of these microbiota-supportive interventions. Our expanding knowledge of gut microbiome, metabolomics and viral immunology will provide a tremendous opportunity to drive microbiota-related discoveries towards clinical applications.

## Author contributions

MW and CL conceived and supervised the project. MW wrote the manuscript and drew the figures. YZ and WC searched and analyzed the literatures. CL and LZ revised the manuscript. All authors contributed to the article and approved the submitted version.
